# Association of Posttraumatic Epilepsy With 1-Year Outcomes After Traumatic Brain Injury

**DOI:** 10.1001/jamanetworkopen.2021.40191

**Published:** 2021-12-29

**Authors:** John Burke, James Gugger, Kan Ding, Jennifer A. Kim, Brandon Foreman, John K. Yue, Ava M. Puccio, Esther L. Yuh, Xiaoying Sun, Miri Rabinowitz, Mary J. Vassar, Sabrina R. Taylor, Ethan A. Winkler, Hansen Deng, Michael McCrea, Murray B. Stein, Claudia S. Robertson, Harvey S. Levin, Sureyya Dikmen, Nancy R. Temkin, Jason Barber, Joseph T. Giacino, Pratik Mukherjee, Kevin K. W. Wang, David O. Okonkwo, Amy J. Markowitz, Sonia Jain, Daniel Lowenstein, Geoffrey T. Manley, Ramon Diaz-Arrastia, and the TRACK-TBI Investigators, Neeraj Badjatia, Ann-Christine Duhaime, V. Ramana Feeser, Etienne Gaudette, Shankar Gopinath, C. Dirk Keene, Frederick K. Korley, Christopher Madden, Randall Merchant, David Schnyer, Ross Zafonte

**Affiliations:** 1 Department of Neurosurgery, University of California, San Francisco; 2 Brain and Spinal Injury Center, Zuckerberg San Francisco General Hospital, San Francisco, California; 3 Department of Neurology, University of Pennsylvania, Philadelphia; 4 Department of Neurology, University of Texas Southwestern Medical Center, Dallas; 5 Department of Neurology, Yale University School of Medicine, New Haven, Connecticut; 6 Department of Neurology and Rehabilitation Medicine, University of Cincinnati, Cincinnati, Ohio; 7 Department of Neurosurgery, University of Pittsburgh Medical Center, Pittsburgh, Pennsylvania; 8 Department of Radiology, University of California. San Francisco; 9 Department of Family Medicine and Public Health, University of California, San Diego; 10 Department of Neurosurgery, Medical College of Wisconsin, Milwaukee; 11 Department of Psychiatry and Public Health, University of California, San Diego; 12 Departments of Neurosurgery and Critical Care, Baylor College of Medicine, Houston, Texas; 13 Departments of Neurosurgery and Neurology, Baylor College of Medicine, Houston, Texas; 14 Department of Rehabilitation Medicine, University of Washington, Seattle; 15 Department of Neurosurgery, University of Washington, Seattle; 16 Departments of Biostatistics, University of Washington, Seattle; 17 Rehabilitation Neuropsychology, Spaulding Rehabilitation Hospital, Harvard Medical School, Boston, Massachusetts; 18 Department of Psychiatry and Neurosciences, McKnight Brain Institute, University of Florida, Gainesville; 19 Department of Neurology, University of California, San Francisco; 20 University of Maryland, Baltimore; 21 MassGeneral Hospital for Children, Boston, Massachusetts; 22 Virginia Commonwealth University, Richmond; 23 University of Toronto, Toronto, Canada; 24 Baylor College of Medicine, Houston, Texas; 25 University of Washington, Seattle; 26 University of Michigan, Ann Arbor; 27 UT Southwestern Medical Center, Dallas, Texas; 28 The University of Texas at Austin, Austin; 29 Harvard Medical School, Boson, Massachusetts

## Abstract

**Importance:**

Posttraumatic epilepsy (PTE) is a recognized sequela of traumatic brain injury (TBI), but the long-term outcomes associated with PTE independent of injury severity are not precisely known.

**Objective:**

To determine the incidence, risk factors, and association with functional outcomes and self-reported somatic, cognitive, and psychological concerns of self-reported PTE in a large, prospectively collected TBI cohort.

**Design, Setting, and Participants:**

This multicenter, prospective cohort study was conducted as part of the Transforming Research and Clinical Knowledge in Traumatic Brain Injury study and identified patients presenting with TBI to 1 of 18 participating level 1 US trauma centers from February 2014 to July 2018. Patients with TBI, extracranial orthopedic injuries (orthopedic controls), and individuals without reported injuries (eg, friends and family of participants; hereafter *friend controls*) were prospectively followed for 12 months. Data were analyzed from January 2020 to April 2021.

**Exposure:**

Demographic, imaging, and clinical information was collected according to TBI Common Data Elements. Incidence of self-reported PTE was assessed using the National Institute of Neurological Disorders and Stroke Epilepsy Screening Questionnaire (NINDS-ESQ).

**Main Outcomes and Measures:**

Primary outcomes included Glasgow Outcome Scale Extended, Rivermead Cognitive Metric (RCM; derived from the Rivermead Post Concussion Symptoms Questionnaire), and the Brief Symptom Inventory-18 (BSI).

**Results:**

Of 3296 participants identified as part of the study, 3044 met inclusion criteria, and 1885 participants (mean [SD] age, 41.3 [17.1] years; 1241 [65.8%] men and 644 [34.2%] women) had follow-up information at 12 months, including 1493 patients with TBI; 182 orthopedic controls, 210 uninjured friend controls; 41 patients with TBI (2.8%) and no controls had positive screening results for PTE. Compared with a negative screening result for PTE, having a positive screening result for PTE was associated with presenting Glasgow Coma Scale score (8.1 [4.8] vs.13.5 [3.3]; *P* < .001) as well as with anomalous acute head imaging findings (risk ratio, 6.42 [95% CI, 2.71-15.22]). After controlling for age, initial Glasgow Coma Scale score, and imaging findings, compared with patients with TBI and without PTE, patients with TBI and with positive PTE screening results had significantly lower Glasgow Outcome Scale Extended scores (mean [SD], 6.1 [1.7] vs 4.7 [1.5]; *P* < .001), higher BSI scores (mean [SD], 50.2 [10.7] vs 58.6 [10.8]; *P* = .02), and higher RCM scores (mean [SD], 3.1 [2.6] vs 5.3 [1.9]; *P* = .002) at 12 months.

**Conclusions and Relevance:**

In this cohort study, the incidence of self-reported PTE after TBI was found to be 2.8% and was independently associated with unfavorable outcomes. These findings highlight the need for effective antiepileptogenic therapies after TBI.

## Key Points

**Question:**

Is posttraumatic epilepsy (PTE) associated with unfavorable functional outcomes after traumatic brain injury (TBI)?

**Findings:**

In this cohort study that included 3296 individuals, the incidence of self-reported diagnosis of PTE at 1 year after injury among individuals who had experienced TBI was 2.8%. Patients with PTE had worse outcome scores at 1 year after injury compared with those without PTE, even after accounting for injury severity and neuroimaging findings on cranial computed tomography.

**Meaning:**

These findings suggest that PTE is a significant sequela of TBI with adverse associations with recovery.

## Teaser

This cohort study assesses the incidence, risk factors, and functional outcomes associated with self-reported posttraumatic epilepsy in patients with traumatic brain injury.

## Introduction

Traumatic brain injuries (TBI) are among the most common maladies affecting humanity.^1^ In the US, there are approximately 2.8 million emergency department (ED) visits each year for TBI, of which approximately 282 000 are admitted to the hospital and approximately 56 000 die,^2^ resulting in an estimated societal cost of $76.5 billion annually.^3^ Patients with TBI can develop late symptomatic seizures, usually more than 7 days after injury, which is referred to as *posttraumatic epilepsy* (PTE).^4^ TBI accounts for approximately 4% of focal epilepsy in the general population and is the leading cause of epilepsy with onset in young adulthood (age 15-24 years).^5^ Epilepsy resulting from brain trauma is often difficult to control with medical therapy, and is the cause of epilepsy in approximately 5% of patients referred to specialized epilepsy centers.^6^

Although previous studies have examined the incidence and sequelae of PTE, there are still several open questions that remain to be resolved. First, the prevalence of PTE after mild TBI (Glasgow Coma Scale [GCS] score, 13-15) has not been addressed in a recent, prospective database, to our knowledge. Of the 2 previous studies large enough to examine PTE after mild injuries, one was in the pre–computed tomography (CT) era,^7^ and the other used an administrative database.^8,9^ Second, previous studies of PTE have not been performed using prospectively collected granular data, limiting the risk factors and outcomes that can be statistically associated with the development of PTE. Third, there is little data regarding whether the development of PTE after TBI is associated with worse outcomes. It has been reported that patients with PTE exhibit reduced mental health outcomes after TBI.^9,10^ However, although prospective data regarding the incidence and risk factors of PTE exist,^11^ relatively few studies investigate how PTE is associated with overall outcomes after TBI.

One challenge in addressing these outstanding issues is that there is not a systematic method of identifying PTE in the TBI population. To overcome this, screening questionnaires have been previously used to identify patients with epilepsy and are useful in situations where access to epilepsy care is limited. Specifically, the National Institutes of Neurological Disorders and Stroke (NINDS) has developed an Epilepsy Screening Questionnaire (ESQ),^12^; however, validating its utility requires a large, prospectively followed TBI cohort.

In this study, we use the Transforming Research and Clinical Knowledge in Traumatic Brain Injury (TRACK-TBI) database and provide the first use of the NINDS-ESQ. TRACK-TBI represents a multicenter prospective data registry with more than 2700 participants with TBI and more than 300 control participants. We administered the NINDS-ESQ to patients at 6- and 12-month follow-ups and compared the rates and outcomes of patients whose screening results for PTE were positive with participants who did not report PTE. Importantly, using the highly granular outcome database, we were able to examine the association of self-reported PTE with outcomes, including global functional outcome, as well as cognitive and emotional metrics of patient recovery. Our aim is to provide evidence of face validity of the NINDS-ESQ by (1) examining the incidence of PTE identified in the TRACK-TBI cohort compared with previously reported incidence of PTE for similar populations; (2) assess whether the risk factors associated with self-reported PTE are similar to risk factors previously associated with PTE risk; and (3) by taking advantage of the large size and prospective nature of the TRACK-TBI cohort, investigate the associations of self-reported PTE with poor functional outcomes and persistent posttraumatic symptoms.

## Methods

This cohort study was approved by the institutional review board of each enrolling site. All participants provided written informed consent. This study is reported following the Strengthening the Reporting of Observational Studies in Epidemiology (STROBE) reporting guideline.

### Participants and Study Design

Patients presenting with TBI (GCS score, 3-15) to 1 of 18 participating level 1 US trauma centers from February 26, 2014, to July 27, 2018, were identified and enrolled prospectively in the TRACK-TBI study, as previously described.^13,14^ Written consent was obtained from participants or their legal authorized representatives. Eligibility criteria included presentation within 24 hours of injury with head trauma warranting clinical evaluation with a noncontrast head CT evaluation based on practice guidelines.^15^ Exclusion criteria for TRACK-TBI, in general, were positive pregnancy test result or known pregnancy, imminent death or current life-threatening disease, incarceration, or evidence of serious psychiatric and neurologic disorders that would interfere with consent or follow-up outcome assessment. Additional exclusion criteria for this study included age younger than 17 years and the presence of a pre-existing epilepsy diagnosis.

Participants with TBI were stratified into 3 clinical groups differentiated by clinical care path: (1) ED and discharged stratum; (2) admission stratum: patients admitted to the hospital but not to the intensive care unit (ICU); and (3) ICU stratum: patients admitted directly from an ED or another hospital to the ICU.

Participants were eligible for inclusion as orthopedic trauma controls (OTC) if they presented with isolated trauma to their limbs, pelvis, and/or ribs and had an Abbreviated Injury Score less than 4 for those body regions. OTC participants were identified and enrolled using the same process as that for patients with TBI, except for the head CT requirement. Participants were ineligible from enrollment as an OTC if they had loss of consciousness, disturbance of consciousness, posttraumatic amnesia or retrograde amnesia, or other clinical findings suggestive of a head injury.

Finally, uninjured control participants (hereafter, *friend controls*) were recruited based on a relationship with a TRACK-TBI participant and the ability to provide informed consent. Friend controls were ineligible for enrollment if they had a history of TBI, concussion, or any traumatic injury causing multiple trauma in the 12 months prior to enrollment. In this analysis, OTC and friend controls were sex- and age-matched to participants with TBI.

### NINDS-ESQ

Administration of the NINDS-ESQ occurred at 6- and 12-month follow-ups for all participants, including participants with TBI, OTCs, and friend controls. The questionnaire consisted of screening items in which the participant had to answer if they had experienced (or were told they experienced) any of the following: item 1a, “Uncontrolled movements of part or all of your body such as twitching, jerking, shaking, or going limp, lasting about 5 minutes or less?”; item 1b, “An unexplained change in mental state or level of awareness; or an episode of “spacing out” which you could not control, lasting about 5 minutes or less?”; and item 1c, “Any other type of repeated unusual attacks or convulsions lasting about 5 minutes or less?” In addition, the patient was asked item 1d, “Has anyone ever told you that you have seizure(s) or epilepsy?”.

If the participant answered yes to any of these first-level screening items, then they were additionally asked the following second-level screening items: item 2a, “Did the most recent seizure(s) occur later than 7 days after the date of the traumatic brain injury?”; item 2b, “Has the participant had seizures or epilepsy prior to the traumatic brain injury?”; item 2c, “Has the participant been diagnosed with epilepsy, a seizure disorder, or a single seizure after the date of the traumatic brain injury diagnosis?”; and item 2d “Have you received medication for seizures or epilepsy?” If participants answered positively to item 2c, they were additionally asked the date of the diagnosis and the person who gave them the diagnosis.

**Figure 1.**
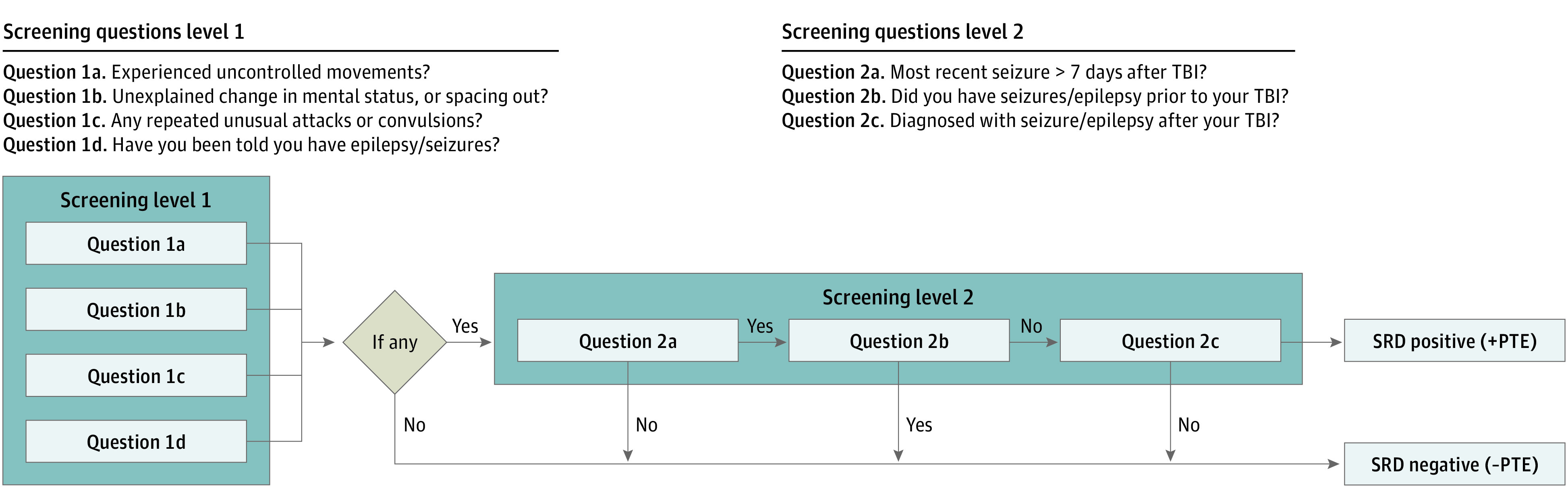
Diagram of Screening Questionnaire and Definition of Self-reported Diagnosis After applying the first-level screening criteria, any patient who screened positive to questions 1a-d was given a second-level screening questionnaire. If the patient answered positively to questions 2a and 2c, and negatively to question 2b they were considered to have a self-reported diagnosis of posttraumatic epilepsy (PTE). SRD indicates self-reported diagnosis; TBI, traumatic brain injury.

For this study, a participant was considered to have a positive self-reported diagnosis of PTE if they answered affirmatively to any first-level screening item (items 1a-1d), and also had seizures that occurred 7 days after their injury (affirmative response to item 2a) and had been given a diagnosis of epilepsy (affirmative response to item 2c). In addition, any participant with a history of epilepsy or seizure disorders found in the historical information collected at the time of injury, or an affirmative response to item 2b was excluded from the analysis (Figure 1). Finally, a positive self-reported diagnosis at 6 months but a negative self-reported diagnosis at 12 months was considered discordant data, not consistent with a feasible response, and likely a misunderstanding of the screening items. Thus, such patients were also excluded from analysis.

### Primary Outcome Measure

Our primary outcome measure was the rate of patients with positive self-reported diagnoses in the TBI vs OTC and FC cohorts. In addition, we also compared several variables between the positive and negative self-reported diagnosis groups, including age, sex, presenting GCS score, presence of loss of consciousness, head CT imaging findings, the presence of hydrocephalus, the Glasgow Outcome Scale Extended (GOSE) score at the 12-month follow up^16^ (focusing on the functional outcomes of the brain injury), the Rivermead Cognitive Metric (RCM) at the 12-month follow up, and the Brief Symptom Inventory-18 (BSI) at the 12-month follow up. Of note, higher scores on the GOSE and RCM reflect better outcomes; however, a higher score on the BSI reflects a worse outcome. Specifically, the GOSE-TBI scale was used, which focuses on the functional limitations from TBI, as opposed to the functional limitations from systemic injuries (ie, GOSE-All).^17^ The RCM was derived from the Rivermead Post Concussion Symptoms Questionnaire (RPQ)^18^ as a linear combination of the subcomponents as follows: 0.76 × [*memory*] + 0.65 × [*concentration*] + 0.76 × [*thinking*]. This linear combination was derived to isolate a purely cognitive metric of the Rivermead Post Concussion Symptoms Questionnaire.^19,20^ Of note, the BSI is a metric of overall mood, and we used the Global Severity Index (GSI), which is a metric based on all 18 items in the BSI.^21^

### Statistical Analysis

Differences in participant characteristics between the positive and negative self-reported diagnosis groups were assessed for statistical significance using Mann-Whitney tests for continuous variables and Fisher exact tests for categorical variables. Differences in outcome among those with TBI were assessed using linear rank-regression adjusting for age (as linear), GCS (mild without hemorrhagic lesions on CT, mild with hemorrhagic lesions on CT, moderate, severe), and CT (negative, hemorrhage, other anomaly). Statistical significance of the 3 primary outcomes (GOSE, BSI, Rivermead Cognitive) was interpreted in the context of multiple comparisons per Benjamini-Hochberg^22^ controlling for a 5% false discovery rate. No adjustments were made for missingness on outcome. Risk ratio involving a zero cell was estimated by first adding 0.5 to all cell counts.^23,24^

Owing to the small number of participants with positive self-reported diagnoses and notable differences compared with the negative self-reported diagnosis group in several injury severity indicators, a sensitivity analysis of outcome was also conducted using a propensity-matched subsample. Propensity scores were generated using a boosted-regression algorithm projecting group membership in either positive or negative self-reported diagnosis groups, based on all demographic, medical history, and injury characteristics analyzed. A maximum matching ratio of 3 controls to each case was used (resulting in an actual mean ratio of approximately 2.6 to 1), using a tolerance caliper of 0.25-fold the pooled SD of the propensity score logits. Only individuals with outcome data were considered for the matching, and separate matching algorithms were run for the GOSE and BSI or Rivermead models.

Two-sample testing and rank-regression modeling were conducted using SPSS statistical software version 26 (IBM). Boosted regression to determine the propensity scores was performed using the TWANG software (RAND). Propensity matching was carried out using the PROC PSMATCH procedure in SAS statistical software version 9.4 (SAS Institute). *P* values were 2-sided, and statistical significance was set at *P* < .05. Data were analyzed from January 2020 to April 2021.

## Results

**Figure 2.**
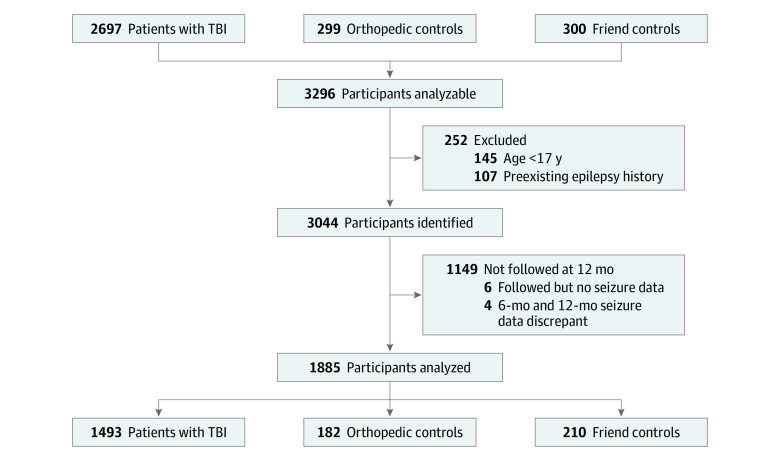
Diagram of Exclusion Criteria and Number of Patients in the Analysis After applying the exclusion criteria, 1493 patients with traumatic brain injury (TBI), 182 orthopedic control patients, and 210 friend control patients were analyzed.

A total of 3296 participants were assessed for eligibility, including 2697 participants with TBI, 299 OTC, and 300 friend controls. Of these, 1885 participants (mean [SD] age, 41.3 [17.1] years; 1241 [65.8%] men and 644 [34.2%] women) met inclusion criteria, had follow-up data at 12 months, and did not have discordant 6- and 12-month responses on the NINDS-ESQ. The final sample included 1493 participants with TBI, 182 OTC, and 210 friend controls (Figure 2).

Table 1 lists the results of the NINDS-ESQ at the 12-month follow-up. All participants who answered positively to any item 1a through 1d were administered level 2 screening items 2a and 2c, which were both required to be positive to establish a positive self-reported diagnosis of PTE. At the 12-month follow-up, 41 participants with TBI (2.7%) had a positive self-reported diagnosis of PTE, and no participants in the OTC or friend control groups had a positive self-reported diagnosis of epilepsy (*P* < .001). Among participants with TBI and self-reported diagnosis, 11 participants (0.9%) had GCS scores of 13 to 15, 3 participants (5.3%) had GCS scores of 9 to 12, and 21 participants (12.2%) had GCS scores 3to 8. The percentage of positive results on screening questions for these 41 patients with positive self-reported diagnosis and the patients without self-reported diagnosis are shown in Table 1.

**Table 1.** Results of NINDS-ESQ at 12-Month Follow-up and Demographic and Imaging Comparison Between Groups

Measure	Self-reported diagnosis, No. (%)		Risk ratio (95% CI)	*P* value
	Negative (n = 1452)	Positive (n = 41)		
**NINDS-ESQ findings**				
Positive: level 1				
1a: Uncontrolled movement				
No	1415 (99.5)	7 (0.5)	NA	NA
Yes	37 (53.6)	32 (46.4)	NA	
Unknown	0	2	NA	
1b: Change in mental state or awareness				
No	1426 (99.2)	12 (0.8)	NA	NA
Yes	24 (48.0)	26 (52.0)	NA	
Unknown	2	3	NA	
1c: Unusual attack				
No	1440 (98.9)	16 (1.1)	NA	NA
Yes	11 (30.6)	25 (69.4)	NA	
Unknown	1	0	NA	
1d: Been told they experienced seizures or epilepsy				
No	1432 (99.8)	3 (0.2)	NA	NA
Yes	19 (33.3)	38 (66.7)	NA	
Unknown	1	0	NA	
Screened positive for level 1				
No	1395 (100)	0	NA	NA
Yes	57 (58.2)	41 (41.8)	NA	
Unknown	1	0		
Positive: level 2				
2a: Seizure 7 d post-TBI				
Not asked (level 1 negative)	1395 (100)	0	NA	NA
No	39 (100)	0	NA	
Yes	12 (22.6)	41 (77.4)	NA	
Unknown	6	0	NA	
2c: Post-TBI epilepsy diagnosis				
Not asked (level 1 negative)	1395 (100)	0	NA	NA
No	49 (100)	0	NA	
Yes	7 (14.6)	41 (85.4)	NA	
Unknown	1	0	NA	
**Participant characteristics**				
Age, mean (SD), y	41.5 (17.6)	35.4 (13.3)	NA	.06
Sex				
Men	974 (97.0)	30 (3.0)	1 [Reference]	.50
Women	478 (97.8)	11 (2.2)	0.75 (0.38-1.49)	
GCS score on ED admission				
Mean (SD)	13.5 (3.3)	8.1 (4.8)	NA	<.001
Median (IQR)	15 (14-15)	7 (3-13.5)	NA	
Severe (3-8)	151 (87.8)	21 (12.2)	15.38 (6.30-37.54)	NA
Moderate (9-12)	54 (94.7)	3 (5.3)	6.63 (1.70-25.8)	NA
Mild (13-15)				
With hemorrhagic lesion on CT	445 (98.9)	5 (1.1)	1.40 (0.43-4.56)	NA
Without hemorrhagic lesion on CT	750 (99.2)	6 (0.8)	1 [Reference]	NA
GCS and CT findings unknown	52	6		
History of mental illness				
No	1122 (97.1)	34 (2.9)	1 [Reference]	.45
Yes	330 (97.9)	7 (2.1)	0.71 (0.32-1.58)	
History of cardiac or pulmonary disease				
No	1015 (96.8)	34 (3.2)	1 [Reference]	.08
Yes	437 (98.4)	7 (1.6)	0.49 (0.22-1.09)	
LOC				
No	167 (12.1)	2 (5.0)	1 [Reference]	.22
Yes	1211 (87.9)	38 (95.0)	2.57 (0.63-10.6)	
Unknown	74	1	NA	
Cause of injury				
Acceleration or deceleration				
No	738 (97.0)	23 (3.0)	1 [Reference]	.53
Yes	713 (97.5)	18 (2.5)	0.81 (0.44-1.50)	
Unknown	1	0	NA	
Crush				
No	1435 (97.2)	41 (2.8)	1 [Reference]	>.99
Yes	16 (100)	0	1.05 (0.07-16.3)^a^	
Unknown	1	0	NA	
Fall from height >1m				
No	1099 (97.2)	32 (2.8)	1 [Reference]	.85
Yes	352 (97.5)	9 (2.5)	0.88 (0.42-1.83)	
Unknown	1	0	NA	
Any high velocity blunt injury				
No	530 (96.4)	20 (3.6)	1 [Reference]	.14
Yes	921 (97.8)	21 (2.2)	0.61 (0.34-1.12)	
Unknown	1	0	NA	
Blow to head				
No	1135 (97.9)	24 (2.1)	1 [Reference]	.007
Yes	316 (94.9)	17 (5.1)	2.47 (1.34-4.53)	
Unknown	1	0	NA	
Head against object				
No	596 (97.7)	14 (2.3)	1 [Reference]	.42
Yes	855 (96.9)	27 (3.1)	1.33 (0.71-2.52)	
Unknown	1	0	NA	
Ground level fall				
No	1215 (97.4)	33 (2.6)	1 [Reference]	.53
Yes	236 (96.7)	8 (3.3)	1.24 (0.58-2.65)	
Unknown	1	0	NA	
Any low velocity blunt injury				
No	290 (99.3)	2 (0.7)	1 [Reference]	.02
Yes	1161 (96.8)	39 (3.3)	4.75 (1.15-19.5)	
Unknown	1	0	NA	
CT showing intracranial injury				
No	777 (99.2)	6 (0.8)	1 [Reference]	<.001
Yes	638 (95.1)	33 (4.9)	6.42 (2.71-15.2)	
Unknown	37	2	NA	
CT result				
Negative	777 (99.2)	6 (0.8)	1 [Reference]	<.001
Positive for intracranial injury without SDH/EDH/IVH	232 (98.7)	3 (1.3)	1.67 (0.42-6.61)	
SDH/EDH/IVH	406 (93.1)	30 (6.9)	8.98 (3.77-21.4)	
Unknown	37	2	NA	
CT SDH				
No	1084 (98.9)	12 (1.1)	1 [Reference]	<.001
Yes	324 (92.6)	26 (7.4)	6.78 (3.46-13.3)	
Unknown	44	3	NA	
CT EDH				
No	1305 (97.9)	28 (2.1)	1 [Reference]	<.001
Yes	103 (91.2)	10 (8.8)	4.21 (2.10-8.45)	
Unknown	44	3	NA	
CT SAH				
No	971 (99.4)	6 (0.6)	1 [Reference]	<.001
Yes	438 (93.2)	32 (6.8)	11.09 (4.67-26.33)	
Unknown	43	3	NA	
CT contusion				
No	1163 (99.0)	12 (1.0)	1 [Reference]	<.001
Yes	245 (90.4)	26 (9.6)	9.39 (4.80-18.4)	
Unknown	44	3	NA	
CT IVH				
No	1359 (97.8)	31 (2.2)	1 [Reference]	.001
Yes	50 (87.7)	7 (12.3)	5.51 (2.53-11.97)	
Unknown	43	3	NA	
CT hydrocephalus				
No	1401 (97.4)	37 (2.6)	1 [Reference]	.17
Yes	6 (85.7)	1 (14.3)	5.55 (0.88-35.04)	
Unknown	43	3	NA	

Abbreviations: CT, computed tomography; EDH, epidural hematoma; GCS, Glasgow Coma Scale; IVH, intraventricular hemorrhage; LOC, loss of consciousness; NA, not applicable; NINDS-ESQ, National Institute of Neurological Disorders and Stroke Epilepsy Screening Questionnaire; SAH, subarachnoid hemorrhage; SDH, subdural hematoma; TBI, traumatic brain injury.

^a^ Risk ratio involving a zero cell was estimated per Pagano and Gauvreau^23^ and Deeks and Higgins^24^ by first adding 0.5 to all cell counts.

As expected, there was a significant association between presenting GCS score and imaging findings, such that the more severe the head injury (ie, the lower the GCS score) the greater the risk of being in the positive self-reported diagnosis group (Table 1). Likewise, any hemorrhage on imaging in the cranial space, whether an epidural hematoma, subdural hematoma, contusion, subarachnoid hemorrhage, or an intraventricular hemorrhage, was associated with an increased chance of a self-reported diagnosis of PTE at 12 months.

We compared 12-month outcomes for the positive and negative self-reported diagnosis groups of participants with TBI (Table 2). The median (IQR) GOSE score was 7 (6-8) for the negative self-reported diagnosis group and 5 (3-6) for the positive self-reported diagnosis group at 12 months (univariate *P* < .001). The mean (SD) BSI was 49.0 (11.5) for the negative self-reported diagnosis group and 56.0 (11.5) for the positive self-reported diagnosis group (univariate *P* = .02). The mean (SD) RCM was 4.9 (2.0) in the positive self-reported diagnosis group, compared with 2.8 (2.6) in the negative self-reported diagnosis group (univariate *P* < .001). The unadjusted differences for the outcome measures are included in Table 2 and were consistent across the entire data set as well as the propensity-matched controls. These results remained significant in the rank-regression analysis adjusted for age, GCS, and head CT findings, as well as in the 3:1 propensity-matched sample. After controlling for age, initial Glasgow Coma Scale score, and imaging findings, compared with patients with TBI and without PTE, patients with TBI and with positive PTE screening results had significantly lower Glasgow Outcome Scale Extended scores (mean [SD], 6.1 [1.7] vs 4.7 [1.5]; *P* < .001), higher BSI scores (mean [SD], 50.2 [10.7] vs 58.6 [10.8]; *P* = .02), and higher RCM scores (mean [SD], 3.1 [2.6] vs 5.3 [1.9]; *P* = .002) at 12 months.

**Table 2.** 12-Month Outcome Analysis According to Self-report Group

	Full sample						3:1 Propensity-matched sample^a^					
	Self-reported PTE diagnosis, No. (%)		Unadjusted difference (SE)	*P* value^b^	*P* value^c^	*P* value^d^	Self-reported PTE diagnosis, No. (%)		Unadjusted difference (SE)	*P* value^b^	*P* value^c^	*P* value^d^
	Negative	Positive					Negative	Positive				
GOSE (TBI)^e^												
Mean (SD)	7.0 (1.2)	4.6 (1.5)	−2.4 (0.2)	<.001	<.001	<.001	6.1 (1.7)	4.7 (1.5)	−1.4 (0.4)	<.001	<.001	<.001
3	34 (2)	16 (39)	NA	NA	NA	NA	10 (13)	11 (35)	NA	NA	NA	NA
4	22 (2)	2 (5)	NA	NA	NA	NA	4 (5)	2 (6)	NA	NA	NA	NA
5	106 (8)	10 (24)	NA	NA	NA	NA	12 (16)	7 (23)	NA	NA	NA	NA
6	231 (17)	9 (22)	NA	NA	NA	NA	14 (18)	7 (23)	NA	NA	NA	NA
7	363 (26)	3 (7)	NA	NA	NA	NA	14 (18)	3 (10)	NA	NA	NA	NA
8	620 (45)	1 (2)	NA	NA	NA	NA	22 (29)	1 (3)	NA	NA	NA	NA
BSI (GSI t-score), mean (SD)^f^	49.0 (11.5)	56.0 (11.5)	7.0 (2.4)	.007	.017	.017	50.2 (10.7)	58.6 (10.8)	8.4 (3.0)	.01	.02	.02
Rivermead (cognitive), mean (SD)^f^^,^^g^	2.8 (2.6)	4.9 (2.0)	2.1 (2.1)	<.001	.001	.001	3.1 (2.6)	5.3 (1.9)	2.2 (2.2)	.001	.001	.002

Abbreviations: BSI, Brief Symptom Inventory; GOSE, Glasgow Outcome Scale Extended; NA, not applicable; TBI, traumatic brain injury.

^a^ Propensity-matching was based only on cases with that outcome (GOSE or BSI/RPQ).

^b^ Unadjusted (Mann-Whitney).

^c^ Rank-regression adjusted for age, GCS (GCS 13-15 with negative CT; GCS 13-15 with positive CT; GCS 9-12; GCS 3-8), CT (hematoma, other pos, neg).

^d^ Further adjusted for multiple comparisons (Benjamini-Hochberg, m = 3).

^e^ Includes 1736 patients with negative self-reported diagnosis and 41 patients with positive self-reported diagnosis.

^f^ Includes 1480 patients with negative self-reported diagnosis and 23 patients with positive self-reported diagnosis.

^g^ Rivermead Cognitive Metric calculated as 0.76 × [*memory*] + 0.65 × [*concentration*] + 0.76 × [*thinking*].^16^

## Discussion

This cohort study reports on the initial validation of the NINDS-ESQ in the TRACK-TBI cohort, confirms that the risk factors for PTE and the overall incidence were comparable with what has been reported in previous studies, and takes advantage of the large, prospectively collected cohort to investigate the associations of self-reported PTE with poor functional outcomes and persistent posttraumatic symptoms. Including orthopedic and friend controls allowed us to additionally verify that no patients with positive self-reported diagnosis were detected in the control groups. Although a formal validation of the NINDS questionnaire would require a criterion standard diagnosis of PTE by an epileptologist, the fact that the incidence of PTE was consistent with historical cohorts and also that there were no incidents of PTE detected in the control groups increases the confidence that our rate of positive self-reported diagnosis reflects the rate of PTE. The differences in outcome between the reported PTE group and the non-PTE group were consistent across propensity-matched groups as well, arguing for the robustness of this finding across statistical approach. Overall, our data suggest that the ESQ is a useful screening tool to identify PTE in clinical practice or research.^25^

The rate of 2.7% of self-reported diagnosis of PTE found in this study is consistent with prior literature. In particular, studies by Annegers and colleagues^7^ and Hauser and colleagues^26^ prospectively followed a retrospectively identified population of patients with TBI and found 5-year risks of developing PTE at 0.7% for GCS score 13 to 15, 1.2% for GCS score 9 to 12, and 10.0% for GCS score 3 to 8. The rates of self-reported diagnosis in this study (a surrogate for PTE) were 0.9% for GCS score 13 to 15, 5.3% for GCS score 9 to 12, and 12.2% for GCS score 3 to 8. These results are consistent with the Annegers et al data^7^ (albeit at earlier follow up times). In addition, the 0.9% risk for patients with GCS score 13 to 15 was further divided into 2.1% risk for patients with hemorrhagic head CT findings and 0.8% risk for patients with nonhemorrhagic head CT findings. Thus, any hemorrhage on head CT imaging was associated with a 2- to 3-fold risk of developing PTE after mild TBI. In addition, there were no patients with positive self-reported diagnosis in the OTC and the FC groups, as expected, given the low rate of incident epilepsy in non–brain injured populations. Collectively, these results suggest that the NINDS-ESQ could be used as a screening tool for PTE in a large population of patients with TBI.

We also report 12-month outcomes for TBI participants with a self-reported diagnosis of PTE compared with those without a self-reported diagnosis. We found an association of self-reported diagnosis status with metrics of overall global outcome (ie, GOSE score). Other studies have examined PTE in a TBI population and found subtle associations with outcomes. For example, a study by Bushnik et al^10^ implemented a retrospective analysis on a TBI Model Systems cohort and found differences between PTE and non-PTE groups on some quality of life metrics but did not report an association with self-reported cognitive variables or GOSE. In addition, a study by Asikainen et al^27^ found an association of PTE with GOS in a prospective study of pediatric patients; however, this result has not been reported in adults. A study by Juengst et al^9^ and another by Walker et al^28^ found a difference in depression and anxiety for patients with PTE but did not report on overall or cognitive outcomes. The potential link between decreased cognitive function and PTE also raises several questions about the nature of this association. In particular, it is unclear if patients with underlying cognitive risk factors or predispositions for epilepsy have a greater propensity to develop PTE after TBI or if developing PTE after TBI leads to decreased cognitive outcomes. Again, a longer-term cohort is required to study this association, which we leave to future investigations.

We found that patients self-reporting a diagnosis of PTE had less favorable functional, cognitive, and emotional outcomes, after accounting for age, GCS on presentation, and cranial CT findings. These data are novel because studies examining outcomes after PTE have typically not shown a major association with overall outcome in adults.^29^ Although there is a high level of evidence that prophylactically treating with antiepileptic drugs in severe TBI does not improve outcome, the very high risk of epilepsy in this patient population suggests that a continued search for antiepileptogenic therapies is warranted.^30,31^

A novel feature of our study is that it included a large number of participants with relatively mild TBI, including many who were discharged from the ED without anomalous cranial CT findings. Most prior studies of PTE have focused on moderate to severe TBI.^22,25,26,27^ While the incidence of PTE in participants with mild TBI (ie, GCS score 13-15 with or without anomalous CT findings) is low, mild TBI accounts for more than 90% of patients presenting to trauma centers with TBI; thus, the risk of PTE after mild TBI is nontrivial. While this risk could be explained by alternative diagnoses that are not specifically adjudicated in this study (eg, psychogenic nonepileptiform seizures), the epidemiological benefits of targeting patients at risk after mild TBI are significant. This finding is consistent with results from epidemiologic studies using Veterans Health Administration administrative data.^8^

### Limitations

This study has some limitations. The principal limitation of this study is that there is no criterion standard against which to compare the results of the NINDS-ESQ data. Thus, it is not possible to definitively find the incidence of PTE in this population, which would require full work-up and diagnosis by an epileptologist, as well as a longer follow-up time. Also, this screening tool is unlikely to be highly specific for epilepsy, and some patients with positive self-reported diagnosis may have psychogenic nonepileptic seizures, which are common in TBI populations and may be misdiagnosed as epilepsy. However, given the consistency of the rate of self-reported diagnosis compared with prior studies, the agreement with known risk factors for PTE, and the associations with outcomes, we feel that self-reported diagnosis status correlates with the true incidence of PTE. We recently launched the TRACK-TBI Epileptogenesis Study, which will extend these findings by prospectively conducting evaluations with epilepsy specialists 5 years after injury, using the DISCOVER structured interview.^32,33^ This study will comprehensively assess the sensitivity, specificity, and predictive value of NINDS-ESQ. Another limitation to this study is that the screening questionnaire could not address the severity of the epilepsy, frequency of seizures, or refractoriness to medical therapy. Since uncontrolled epilepsy is known to correlate with poor quality of life, future studies should determine whether certain subsets of patients are more affected by seizures after PTE. Additionally, the follow-up rate after TBI in this study was relatively low compared with other disease processes. However, the low rate of follow-up after TBI, especially in large level 1 trauma centers, was expected and should not bias the rate of PTE in the reported patient population.

## Conclusions

This cohort study assessing a screening questionnaire found that the incidence of PTE after TBI was 2.7% at 12 months after injury. Because PTE is associated with unfavorable outcomes, clinicians should have a high degree of suspicion for PTE after TBI and consider antiepileptogenic therapies as needed.
